# Efficacy of an HSV-1 Neuro-Attenuated Vaccine in Mice Is Reduced by Preventing Viral DNA Replication

**DOI:** 10.3390/v14050869

**Published:** 2022-04-22

**Authors:** Hong Wang, David J. Davido, Heba H. Mostafa, Lynda A. Morrison

**Affiliations:** 1 Department of Molecular Microbiology and Immunology, Saint Louis University School of Medicine, St. Louis, MO 63104, USA; wangh@neuro.wustl.edu; 2 Department of Molecular Biosciences, University of Kansas, Lawrence, KS 66045, USA; ddavido@ku.edu (D.J.D.); hmostaf2@jhmi.edu (H.H.M.); 3 Department of Internal Medicine, Saint Louis University School of Medicine, St. Louis, MO 63104, USA

**Keywords:** herpes simplex virus 1, vaccine, corneal infection, replication-defective

## Abstract

We previously isolated an HSV-1 mutant, KOS-NA, that contains two non-synonymous mutations in UL39. One of the mutations, resulting in an R950H amino acid substitution in ICP6, renders KOS-NA severely neuro-attenuated and significantly reduces HSV-1 latency. Vaccination of mice with KOS-NA prior to corneal challenge provides significant protection against HSV-1-mediated eye diseases even at a very low immunizing dose, indicating its utility as a vaccine scaffold. Because KOS-NA contains a neuro-attenuating mutation in a single gene, we sought to improve its safety by deleting a portion of the UL29 gene whose protein product, ICP8, is essential for viral DNA replication. Whereas KOS-NA reduced replication of HSV-1 challenge virus in the corneal epithelium and protected mice against blepharitis and keratitis induced by the challenge virus, KOS-NA/8- and an ICP8- virus were significantly less efficacious except at higher doses. Our results suggest that the capacity to replicate, even at significantly reduced levels compared with wild-type HSV-1, may be an important feature of an effective vaccine. Means to improve safety of attenuated viruses as vaccines without compromising efficacy should be sought.

## 1. Introduction

Infections with herpes simplex virus 1 (HSV-1) can occur at several epithelial sites, causing diseases that include orofacial and genital sores and corneal lesions [1]. HSV-1 establishes latency in sensory neurons innervating the epithelial surface where primary infection has occurred. The virus’ capacity to subsequently reactivate under stress stimuli leads to frequent periods of recurrent infection with HSV-1 [1]. Recurrent infections of the eye after reactivation from the site of latency in the trigeminal ganglion (TG) can lead to corneal scarring and associated herpes stromal keratitis (HSK), a site-threatening condition that afflicts more than 450,000 persons annually in the United States alone [2]. Notably, recurrent bouts of HSK are a principal cause of non-traumatic corneal blindness [3]. Although treatment with antiviral drugs such as acyclovir can limit the severity of orofacial infections, antiviral therapy can reduce time to healing in many patients but does not completely assist patients in resolving recurrent HSK and its associated pathology [4,5,6]. A prophylactic vaccine to prevent or limit HSV-1 ocular infection could dramatically reduce the incidence and severity of HSK [7].

Protein subunit vaccines, which are very safe, are also very effective in protecting against some virus infections such as hepatitis B virus. For other more intransigent viruses, vaccines must tread the line between safety and efficacy. In a phase 3 trial, a subunit vaccine composed of HSV-2 glycoprotein D in adjuvant provided 35% protection of subjects from HSV-1 oral or genital infection and disease [8]. Combinations of two to seven HSV proteins have shown promise in mice and guinea pigs [9,10,11,12,13,14]. Replication-incompetent and single-cycle mutants have similarly proven protective in animal models [15,16] and a replication-defective HSV-2 has successfully passed phase 1 clinical trials [17]. Attenuated, replication-competent HSV vaccines are also being produced and tested [18,19,20,21,22,23]. Modifying replication-competent virus enough to ensure safety as a vaccine is a key consideration.

We isolated an HSV-1 variant (termed KOS-NA) that contains several non-synonymous point mutations in the UL39, US7 and RS1 genes [24]. KOS-NA is reduced for replication in the cornea and trigeminal ganglia of ocularly infected mice and shows diminished HSV-1 latent genome load and reactivation. Genetic studies confirmed that a mutant form of ICP6, the large subunit of ribonucleotide reductase, expressed by KOS-NA is responsible for its attenuated phenotype. The profound neuro-attenuation of KOS-NA suggested its utility as a vaccine, and indeed, it elicited higher titer antibody and IFNγ-producing CD4 and CD8 T cell responses than ICP8- or ICP0- viruses and was efficacious in protecting against HSV-1-induced keratitis and blepharitis in mice [25]. Despite being attenuated for replication in the periphery and nervous system, the immune responses stimulated by KOS-NA and the degree of protection achieved were comparable to the wild-type KOS strain [25]. A point mutation, however neuro-attenuating, would not provide sufficient safety to advance as a vaccine candidate. Therefore, we combined the two vaccine strategies to determine whether the efficacy of KOS-NA as a vaccine could be maintained if KOS-NA is rendered replication-incompetent by incorporating a UL29 mutation (ICP8- virus).

## 2. Materials and Methods

### 2.1. Ethics Statement

This study was carried out in strict accordance with the recommendations in the Guide for the Care and Use of Laboratory Animals of the National Institutes of Health. The protocol and study were approved by the Committee on the Care and Use of Animals of Saint Louis University (NIH assurance number A3225-01; Institutional protocol number 2405). All procedures were conducted in a manner to minimize suffering.

### 2.2. Virus Strains and Replication-Defective Virus Construction

The KOS-NA mutant of HSV-1 KOS [25] contains non-synonymous mutations in the UL39 gene, resulting in L393P and R950H substitutions in ICP6, as well as three additional mutations in US7 and RS1 [24]. To create KOS-NA/8-, the plasmid p8ΔNotI, which bears a 2001 bp NotI-NotI deletion in the UL29 open reading frame [26], was co-transfected with full-length KOS-NA genomic DNA by Amaxa nucleofection (Lonza, Walkersville, MD, USA). To support growth of successfully recombined viral genomes, we used S2 cells, a Vero cell line stably expressing ICP8 [26]. Viruses derived from the co-transfection were collected and distributed into 24-well plates by limiting dilution. Wells containing only one virus plaque per well were screened by PCR for the presence or absence of the NotI region based on differential amplicon size. Plaques containing the deletion were reisolated and confirmed by further PCR screening. A recombinant virus was selected and designated KOS-NA/8-. A parental ICP8- virus, d301 [26], and the KOS-NA viruses were used for comparison. Titer of HSV in virus stocks was quantified by standard plaque assay [27].

### 2.3. Mice

Female BALB/c mice (H-2d) were purchased from the National Cancer Institute (Frederick, MD). All mice were housed at Saint Louis University under specific pathogen-free conditions and were vaccinated at 6 weeks of age.

### 2.4. Immunization and Challenge

Mice were immunized subcutaneously (s.c.) in the hind flank with a low dose (2 × 10^4^ pfu), medium dose (1 × 10^5^ pfu) or high dose (5 × 10^5^ pfu) of HSV-1 vaccine strains in the KOS background. Twenty-eight days after immunization, mice were deeply anesthetized, their corneas were scarified, and they were challenged by dropwise application of 4 × 10^5^ pfu/eye HSV-1 strain mP.

### 2.5. Post-Challenge Assessments

Virus replication in the corneal epithelium was assessed by gently swabbing eyes with moistened cotton-tipped swabs at 4 h and days 1 through 4 post-infection. Swabs for each mouse were placed together in 1 mL PBS and frozen at −80 °C until assay. Virus yield was quantified on Vero cell monolayers by standard plaque assay. Daily monitoring of body weight, signs of blepharitis and survival was conducted after challenge. Mice were weighed individually and mean change from initial body weight was calculated daily for each group. Blepharitis scores were assigned in masked fashion based on the following scale: 0, no apparent signs of disease; 1, mild swelling and erythema of the eyelid; 2, moderate swelling and crusty exudate; 3, periocular lesions and depilation; and 4, extensive lesions and depilation. Mean daily disease score was calculated for each group. Keratitis was assessed at 9 d post-challenge using an ophthalmoscope and scored in masked fashion based on the following scale: 0, no apparent signs of disease; 1, mild opacity; 2 moderate opacity with discernible iris features; 3, dense opacity; 4, dense opacity with ulceration.

### 2.6. Quantitation of Serum Antibodies

To determine the titer of HSV-specific serum antibodies induced by vaccine, mice were immunized with vaccine virus or control supernatant. Blood was collected from the tail vein of mice 21 d after immunization. Serum was prepared by clot retraction and analyzed by ELISA as previously described [28]. Anti-mouse-IgG-biotin (R & D Systems, Minneapolis, MN, USA) was used as secondary antibody and detected using streptavidin-HRP followed by OPD substrate (Sigma-Aldrich, St. Louis, MO, USA). Alternatively, antimouse-IgG1-HRP and -IgG2a-HRP (SouthernBiotech, Birmingham, AL, USA) were used. Plates were read at 490 nm on a BioRad 680 reader. Antibody concentrations were determined by comparison to standard curves generated with serum containing known concentrations of IgG captured on plates coated with goat-anti-kappa light chain antibody (Caltag, Burlingame, CA, USA) as previously described [28].

### 2.7. Statistical Analyses

Significance of difference in virus titers in the cornea on individual days was determined by one way analysis of variance (ANOVA) with Dunnett’s post-hoc test for multiple comparisons. The Kruskal-Wallis non-parametric test with Dunn’s test was used to assess the significance of difference in blepharitis scores on individual days post-challenge. Differences in keratitis scores were compared using ANOVA with Dunnett’s post-hoc test. Significance differences with body weight and antibody titers were performed using one way ANOVA. Analysis for survival studies was performed using the log-rank test.

## 3. Results

### 3.1. Construction of KOS-NA/8-

The KOS-NA mutant of HSV-1 KOS [25] has a non-synonymous mutation in the UL39 gene, which produces an R950H substitution in ICP6 responsible for the KOS-NA phenotypes [24]. To construct an ICP8-, replication-incompetent version of KOS-NA, a 2 kb fragment of ICP8 was excised from the UL29 gene of strain KOS in plasmid p8BS, and the plasmid DNA was co-transfected into ICP8-expressing cells along with full length KOS-NA DNA. Plaque isolates were screened by PCR and a recombinant virus containing the deletion in UL29 was identified and purified. The new, replication-incompetent mutant, termed KOS-NA/8-, was compared with the parental KOS-NA strain, and an ICP8- strain, d301, for capacity to protect mice as a vaccine. Groups of BALB/c mice were immunized s.c. with a single dose of a vaccine strain, either low (2 × 10^4^ pfu), medium (1 × 10^5^ pfu) or high (5 × 10^5^ pfu). Supernatant from uninfected cells was used as a control. One month after vaccination, mice were challenged by inoculation onto the scarified corneas of 4 × 10^5^ pfu/eye of heterologous HSV-1 strain mP.

### 3.2. Effect of Vaccine Strains on Challenge Virus Replication in the Cornea

**Figure 1:**
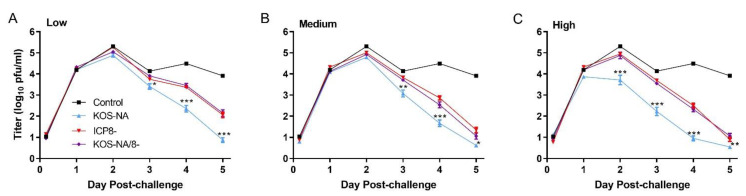
Eye titers of challenge virus from immunized mice. Groups of 10 BALB/c mice were vaccinated with medium (control) or (**A**) low (2 × 10^4^ pfu), (**B**) medium (1 × 10^5^ pfu), or (**C**) high (5 × 10^5^ pfu) doses of KOS-NA, ICP8-, and KOS-NA/8-. The control group is the same for all three graphs. All groups were challenged with HSV-1 strain mP (4 × 10^5^ pfu/eye), 30 days post-immunization. Eye swabs were taken at the times indicated (0–5 days post-infection), and titers were determined by standard plaque assays. Data represent the geometric means ± SEM from 3 independent experiments combined for *n* = 30 mice per group. *** *p* < 0.0001; ** *p* = 0.0002–0.0008; and * *p* = 0.0011–0.0064 for KOS-NA compared with ICP8- and KOS-NA/8- viruses.

Virus titer was determined in tear film collected at 4 h and days 1 to 5 post-challenge. At the low immunizing dose, KOS-NA significantly reduced challenge virus replication beginning 3 d post-challenge compared with control supernatant and ICP8- and KOS-NA/8- vaccines (Figure 1A). A similar picture emerged in mice receiving the medium dose of vaccine, except that the effect of prior vaccination appeared to be more profound and the replication-incompetent vaccines had a greater suppressive effect than at the low dose (Figure 1B). At the high vaccine dose, KOS-NA further decreased challenge virus replication beginning 2 d post-challenge (Figure 1C). The replication-incompetent viruses KOS-NA/8- and ICP8- closely resembled each other at all vaccine doses and time points examined and impacted the amount of challenge virus shed from the cornea only at 4 and 5 d post-challenge relative to the control supernatant (Figure 1).

### 3.3. Effect of Vaccine Strains on Eye Diseases

**Figure 2:**
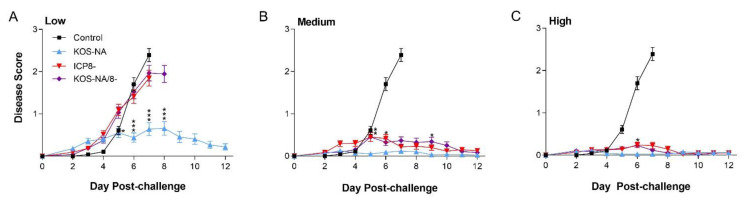
Protection of mice from blepharitis after corneal challenge. Groups of 10 mice immunized with (**A**) low (2 × 10^4^ pfu), (**B**) medium (1 × 10^5^ pfu), or (**C**) high (5 × 10^5^ pfu) doses of the indicated viruses and 1 group of mice immunized with supernatant (control) were challenged by corneal infection with HSV-1 strain mP (4 × 10^5^ pfu/eye) and scored daily for signs of eyelid disease. Values are the mean ± SEM score of eyelids from 3 independent experiments combined for *n* = 30 mice. *** *p* < 0.0001; ** *p* = 0.0002-0.0003; * *p* = 0.0011–0.0092 note significant differences comparing KOS-NA to ICP8- and/or KOS-NA/8-.

Disease of the eyelid (blepharitis) was assessed in masked fashion for nearly 2 weeks post-challenge. In mice immunized with the low vaccine dose (Figure 2A), only KOS-NA clearly protected against blepharitis after 5 d post-challenge, the point at which mice immunized with control supernatant began to develop severe signs of disease (Figure 2B). At the medium dose, however, KOS-NA/8- and ICP8- vaccines also protected mice from developing severe disease (Figure 2B). Even so, KOS-NA provided better protection than the replication-incompetent vaccine strains at several time points. The high dose of vaccine effectively protected mice from blepharitis whether the vaccine was replication-competent or not (Figure 2C).

**Figure 3:**
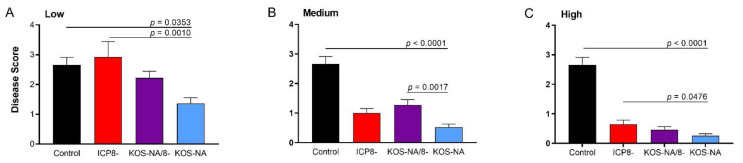
Protection of mice from severe keratitis after corneal challenge. The corneas of the same groups of mice described in Figure 2 were evaluated at 9 days post-challenge for signs of keratitis at (**A**) low (2 × 10^4^ pfu), (**B**) medium (1 × 10^5^ pfu), or (**C**) high (5 × 10^5^ pfu) vaccine doses. Values represent the mean disease scores ± SEM compiled from 3 independent experiments; *n* = 11 to 30 mice examined per group. *p*-values shown were determined by one way ANOVA with Dunnett’s correction for multiple groups compared to KOS-NA.

Corneas of mice were examined by a masked observer 9 d post-challenge. Keratitis was evident in most eyes of control mice (Figure 3A). At the low vaccine dose, mice immunized with KOS-NA/8- or ICP8- developed as much corneal disease as control mice; those immunized with KOS-NA had less corneal disease compared with the control and ICP8- vaccine groups (Figure 3A). At the medium vaccine dose, all three vaccine strains provided significant protection of the cornea compared with the control mice. In addition, KOS-NA further suppressed development of keratitis compared with KOS-NA/8- (Figure 3B). Mice receiving a single high dose of any vaccine strain were well-protected against keratitis, though again, KOS-NA proved superior to the replication-incompetent ICP8- vaccine (Figure 3C), at least by this qualitative measure of eye disease.

### 3.4. Effect of Vaccine Strains on Health and Survival

**Figure 4:**
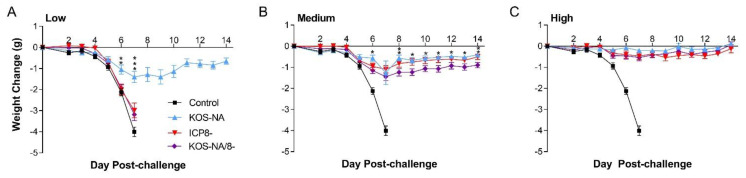
Body weight change upon virus challenge. Groups of 10 BALB/c mice were vaccinated with supernatant (control) or (**A**) low (2 × 10^4^ pfu), (**B**) medium (1 × 10^5^ pfu), or (**C**) high (5 × 10^5^ pfu) doses of KOS-NA, ICP8-, and KOS-NA/8-. At 30 days post-vaccination, mice were weighed and challenged with HSV-1 strain mP (4 × 10^5^ pfu/eye) by corneal infection. Mice were then weighed daily post-challenge. Data shown are the mean weight change per vaccination group ± SEM from 3 independent experiments combined for *n* = 30 mice per group. *** *p* < 0.0004; ** *p* = 0.0032–0.0089; * *p* = 0.012–0.030 represent significant differences between KOS-NA relative to ICP8- and/or KOS-NA/8- as determined by one way ANOVA with Dunnett’s correction for multiple groups.

Body weight of the mice was determined for 2 weeks post-challenge as a sensitive indicator of general health. At the low vaccine dose, only KOS-NA reduced weight loss post-challenge (Figure 4A). Mice immunized with the medium dose of any vaccine experienced little change in body weight compared with the control mice, although interestingly, mice immunized with KOS-NA/8- were slightly less protected than KOS-NA (Figure 4B). All vaccines given at the high dosage completely protected mice from weight loss (Figure 4C).

**Figure 5:**
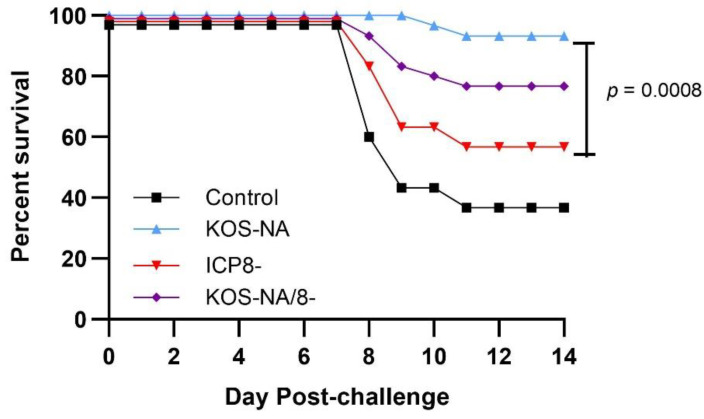
Survival of vaccinated mice after virus challenge. Mice were vaccinated with supernatant (control) or a low (2 × 10^4^ pfu/eye) dose of KOS-NA, ICP8-, and KOS-NA/8-. At 30 days post-vaccination, mice were challenged with HSV-1 strain mP (4 × 10^5^ pfu/eye) by corneal infection, and their survival was observed up to 14 days post-challenge. Data shown are survival ± SEM from 3 independent experiments. Survival of KOS-NA-vaccinated mice was statistically different from ICP8- by the log-rank test.

Only mice immunized with control supernatant or the low dose of vaccine viruses experienced symptoms severe enough to be lethal. Nearly all mice immunized with KOS-NA survived challenge (Figure 5). Mice immunized with a replication-incompetent vaccine survived less frequently than KOS-NA but were better protected than control mice, indicating some level of antiviral immunity that protected against neurovirulence was still generated.

### 3.5. Antibody Response to Immunization

**Figure 6:**
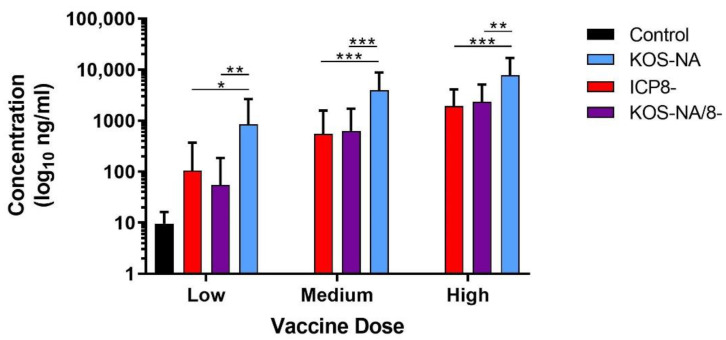
Titers of HSV-specific antibody in immunized mice. Groups of 10 BALB/c mice were immunized with low (2 × 10^4^ pfu), medium (1 × 10^5^ pfu), or high (5 × 10^5^ pfu) doses of the indicated viruses, and 1 group of mice was immunized with supernatant (control) as a negative control. Blood was collected 21 days post-immunization, and HSV-specific serum IgG was quantified by ELISA. Data represent the geometric mean titer for each group ± SEM and are the combined results of 3 independent experiments for *n* = 30 mice per group. *** *p* < 0.0004, ** *p* = 0.0011–0.0055, and * *p* = 0.011 indicate significant differences for KOS-NA compared to ICP8- and KOS-NA/8- viruses using one way ANOVA.

Serum had been collected from the mice 3 weeks after immunization. When analyzed for HSV-specific antibody by ELISA, it was evident that both replication-incompetent strains provoked a humoral response inferior to the replication-competent KOS-NA vaccine regardless of the immunizing dose (Figure 6). The antibody response was dose-dependent and a high dose of KOS-NA/8- was needed to achieve similar titers as a low dose of KOS-NA. Thus, deletion of a gene essential for virus replication diminishes the stimulus for antiviral immunoglobin production. This result may in part explain the reduced efficiency of replication-incompetent vaccine virus, but assays for neutralization and antibody-dependent cellular cytotoxicity will be needed to determine functional deficit(s).

## 4. Discussion

KOS-NA impaired the establishment of latency against challenge virus as a vaccine and proved as immunogenic and protective as wild-type virus, despite being attenuated for replication in the periphery [25], an advantageous characteristic for a potential vaccine. We attempted to combine this mutation with one rendering the virus replication-incompetent to determine whether protective capacity could be maintained while increasing safety of KOS-NA. Our results demonstrate that the principal determinant of enhanced protection is the capacity of KOS-NA to replicate; by all measures, KOS-NA/8- was not different from an ICP8- virus when used as a vaccine in mice. The effects on individual parameters of disease were dose dependent, such that a higher dose of replication-incompetent vaccine achieved parity with a lower dose of KOS-NA. Interestingly, a virus containing a UL41 mutation is immunogenic and protective against HSV-1-induced corneal disease [29], and we have shown that the UL41 mutation combined with deletion of ICP8 causes only moderate attenuation of vaccine efficacy [30]. These results suggest that replication competence is an important consideration when designing efficient virus vaccines against HSV-1, but combinations of genetic defects must be assessed individually.

Testing vaccine strains over a range of doses is extremely important because various measures of vaccine efficacy are differentially affected by vaccine dose. For example, we discerned KOS-NA’s superior protective effect against replication in the eye at any dose; however, significant differences between KOS-NA and replication-incompetent viruses in their effects on body weight, blepharitis and keratitis were most apparent in mice immunized with the low vaccine dose. Similarly, 5-fold more replication-incompetent vaccine was required to offer the same level of protection against weight loss, blepharitis and keratitis as the low dose of KOS-NA, whereas at least 10-fold more replication-incompetent vaccine was required to reduce replication in the cornea equivalently.

Our results demonstrate that replication-incompetent vaccines, although more stimulatory than glycoprotein subunit vaccines [31,32], may require further modification to improve their immunogenicity if a lower dose of vaccine is a desired goal [33]. ICP8- viruses still express immediate-early, early, and delayed-early (γ1) proteins [26], so then why did we lose vaccine efficacy with KOS-NA/8- compared with KOS-NA? There are several possibilities that may explain the result. HSV true late (γ2) proteins, virion synthesis, or both may induce components of the immune response to control viral infection and disease in KOS-NA that are not present in KOS-NA/8-. Monitoring of antibody and T cell responses to a γ2 protein could help answer this question. Another plausible explanation is that some amplification of viral protein expression is helpful in reaching a stimulation threshold for robust immunity. This might also be accomplished by deleting UL41 [30], co-expressing B7 co-stimulation molecules [33], giving a larger vaccine dose (as suggested by this study), deleting an essential γ2 gene that does not affect overall viral protein expression in a single infectious cycle, and/or providing a booster vaccination. Future studies will be directed toward altering KOS-NA by mutation of genes involved in pathogenesis or immune evasion, rendering it safer.

HSK develops as a result of the immune response in the cornea subsequent to repeated reactivations [34]. Thus, a vaccine to prevent eye disease caused by HSV-1 also must elicit protective rather than pathological immune responses. KOS-NA and KOS-NA/8- each protected mice from developing blepharitis and keratitis after corneal challenge, at first pass appearing to fulfill this vaccine requirement. We previously demonstrated that KOS-NA elicits a strong HSV-specific antibody response and memory CD4 and CD8 T cells [25]. Once KOS-NA is modified by incorporation of additional mutation(s) to confer safety without compromising efficacy, more in-depth analyses of the immune responses to vaccination are warranted.

## Data Availability

The data presented in this study are available on request from the corresponding author.

## Abbreviations

HSV-1	herpes simplex virus 1
ICP8	infected cell protein 8
Pfu	plaque-forming units
